# Household secondhand smoke exposure and learning difficulties in children and adolescents: A cross-sectional study on the mediating roles of attention, memory, and adaptability

**DOI:** 10.18332/tid/226713

**Published:** 2026-10-31

**Authors:** Zihua Lu, Baisen Ye

**Affiliations:** 1 Yangzhou University, Yangzhou, China; 2 Yangzhou Polytechnic University, Yangzhou, China

**Keywords:** household secondhand smoke exposure, learning difficulties, cognition, NHANES, child development

## Abstract

**INTRODUCTION:**

Secondhand smoke exposure is a prevalent and modifiable factor linked to adverse neurodevelopmental outcomes in children and adolescents. However, the mechanisms through which household smoking contributes to learning difficulties remain unclear. Therefore, this study aimed to investigate the association between secondhand smoke exposure and learning difficulties in a nationally representative sample of children and adolescents, and to examine the potential mediating roles of attention difficulties, memory difficulties, and difficulties accepting change.

**METHODS:**

This pooled secondary data analysis used National Health and Nutrition Examination Survey (NHANES, USA) 2021–2023 data from 2455 participants aged 5–17 years. The exposure was household smoking status; the outcome was parent/self-reported learning difficulties; and potential mediators were attention, memory, and adaptability difficulties. Using weighted multivariable logistic regression, we adjusted for demographics, poverty income ratio, anxiety, and depression. Mediation effects were quantified via the Sobel test.

**RESULTS:**

The prevalence of learning difficulties was significantly higher in children from households with smokers (24.6%) compared to those without (17.4%, OR=1.54; 95% CI: 1.24–1.92, p=0.002). After full adjustment for covariates, secondhand smoke exposure remained significantly associated with increased odds of learning difficulties (adjusted odds ratio, AOR=1.43; 95% CI: 1.14–1.79, p=0.002). Mediation analysis confirmed significant indirect effects through memory difficulties (52.1% mediated; β=0.192; 95% CI: 0.055–0.335, p=0.006), attention difficulties (44.3%; β=0.164; 95% CI: 0.070–0.265, p<0.001), and difficulties accepting change (36.4%; β=0.135; 95% CI: 0.074–0.199, p<0.001).

**CONCLUSIONS:**

Household secondhand smoke exposure is independently associated with learning difficulties in US children and adolescents. This association is predominantly mediated through impairments in core cognitive functions, particularly attention, memory, and adaptability.

## INTRODUCTION

Learning difficulties in children and adolescents represent a complex public health issue, with etiology involving the interaction of multiple factors including genetic, environmental, and psychosocial elements^1^. Among various environmental risk factors, secondhand smoke exposure, being both prevalent and modifiable, has garnered significant attention^2^. Numerous epidemiological studies have confirmed that both prenatal tobacco smoke (PTS) and postnatal secondhand smoke exert significant negative effects on neurodevelopment and academic achievement in children and adolescents^3,4^. For instance, a cross-sectional study of Brazilian schoolchildren revealed that having household members who smoke in the child’s presence was associated with more than a sixfold higher odds of learning difficulties (prevalence ratio=6.1), highlighting the severe harm of a home smoking environment^2^.

Although the association between household smoking and learning difficulties has been preliminarily established, the mediating mechanisms – how tobacco smoke affects the learning ability of children and adolescents – remains a core question requiring in-depth exploration. Existing evidence suggests that impaired cognitive function may be a key pathway linking secondhand smoke exposure to learning outcomes^5^. Nicotine, as a neuroactive substance, can significantly affect cognitive processes, including attention, working memory, and episodic memory, by acting on nicotinic acetylcholine receptors (nAChRs) in the brain, particularly those containing α4, β2, and α7 subunits^6^. Empirical research supports this: a Korean study found that children’s TSE, measured using urinary cotinine as a biomarker, was significantly associated with more omission errors, commission errors, and greater reaction time variability on the Continuous Performance Test (CPT)^7^. Furthermore, these attention metrics mediated the relationship between secondhand smoke exposure and learning difficulty symptoms, strongly suggesting that attentional function may be an important mediating variable.

Additionally, memory, particularly verbal learning and memory ability, has also been found to be vulnerable to impairment from secondhand smoke exposure^8^. A study of university students in Yemen showed that individuals who both chewed khat and smoked tobacco performed significantly worse on word learning and delayed recall tests compared to controls who only chewed khat or used no substances, indicating that tobacco use is associated with slowed verbal learning and memory retrieval^9^. More broadly, secondhand smoke exposure is closely linked to a range of neurodevelopmental issues. Analysis of data from the National Survey of Children’s Health (NSCH) in the United States indicated that children living in households with smokers (regardless of whether smoking occurred indoors) had significantly elevated risks of attention-deficit/hyperactivity disorder (ADHD), behavioral problems, anxiety, depression, and learning disabilities, with this association being more pronounced in preschool-aged children^10,11^.

However, gaps remain in the existing research. First, most studies focus on the mediating role of a single cognitive domain, with few systematic evaluations of the joint mediating effects of multiple core cognitive functions within a unified framework. Second, studies utilizing large-scale, nationally representative population data with complex sampling design to examine these mediating pathways are still insufficient^2,7^. The latest data from the 2021–2023 cycle of NHANES, one of the most representative health surveys in the US^12^, provide a valuable opportunity to address these gaps.

Therefore, this study aims to utilize the latest NHANES 2021–2023 data to thoroughly explore the association between household secondhand smoke exposure and learning difficulties in a nationally representative sample of children and adolescents aged 5–17 years, and to investigate the potential mediating roles of three key cognitive dimensions: attention difficulties, memory difficulties, and difficulties accepting change.

## METHODS

### Data source and study population

This pooled secondary data analysis is based on a nationally representative sample of the US population. The National Health and Nutrition Examination Survey (NHANES 2021–2023), conducted by the National Center for Health Statistics, is a cross-sectional covering the period from August 2021 to August 2023^13^. As the learning difficulty questionnaire was administered only to individuals aged 5–17 years, the study population was restricted to this age group. Initial screening identified 2789 respondents aged 5–17 years. After excluding 344 individuals with missing data on the two primary variables (household smoking and learning difficulties), the final analytical sample comprised 2455 children and adolescents.

### Main variable questionnaires

The core independent variable was household secondhand smoke exposure, derived from the questionnaire item: ‘How many people who live here smoke tobacco?’. The original variable had three categories: 0 (‘No one in household is a smoker’), 1 (‘1 household member is a smoker’), and 2 (‘2 or more household members are smokers’). Among the 2455 participants included, those with ≥2 smokers represented a small proportion of the sample. The rationale for merging categories 1 and 2 into a single binary variable (‘Has smoker(s)’ vs ‘No smoker’) was twofold: first, to ensure sufficient statistical power and model stability for subsequent multivariate adjusted models and subgroup analyses by avoiding extremely small cell sizes in the category with ≥2 smokers; second, based on the primary research objective to estimate the overall effect of exposure versus non-exposure to a household secondhand smoke exposure, rather than to precisely quantify a dose-response relationship.(all NHANES variable names used in this study are listed in Supplementary file Table S1).

The outcome variable was learning difficulty, based on self- or proxy-reported data from the question: ‘Compared with children of the same age, do you/does the sample person (SP) have difficulty learning things?’. Original response options (‘No difficulty’, ‘Some difficulty’, ‘A lot of difficulty’, and ‘Cannot do at all’) were dichotomized into ‘No difficulty’ versus ‘Any difficulty’ due to the small cell counts in the most severe response categories. To ensure the robustness of the statistical analysis and the interpretability of the results, these three categories were combined into a single ‘Has difficulty’ group, creating a binary variable relative to ‘No difficulty’. This approach preserves the essential characteristic of the core concept while ensuring stability in parameter estimation for subsequent complex statistical models.

### Covariates

Covariates included gender (male, female), age, and race/ethnicity (Mexican American, Non-Hispanic Black, Non-Hispanic White, Other Hispanic, Other race), representing personal characteristics; poverty income ratio (PIR; household economic status); and two student mental health variables: anxiety (yes, no) and depression (yes, no), all established factors associated with learning difficulties^14^. Anxiety was assessed by the question: ‘How often is SP/are you very anxious, nervous, or worried?’. Depression was assessed by: ‘How often is SP/are you very sad or depressed?’. Responses for both were: daily, weekly, monthly, a few times a year, never (coded 1–5). Responses from ‘daily’ to ‘a few times a year’ were combined into ‘yes’, and ‘never’ was defined as ‘no’.

Three cognitive function variables were assessed: difficulty remembering things, difficulty concentrating, and difficulty accepting change. Similar to learning difficulty, these originally had four response levels (‘no difficulty’ to ‘cannot do at all’), and the last three were combined into ‘yes’ for analysis.

### Statistical analysis

*Descriptive analyses*

Descriptive analyses accounted for NHANES’s complex survey design (stratification, clustering, sampling weights). To characterize the study population and assess the baseline distribution of potential confounding variables across exposure groups, Weighted chi-squared tests and weighted ANOVA were used to compare distributions of demographic characteristics, socioeconomic factors, cognitive function indicators, and psychological/behavioral variables between different household smoking groups. Additionally, a parallel descriptive analysis was performed, stratifying participants by the outcome variable (learning difficulty status) to examine the distribution of all covariates, including the primary exposure. Analyses were conducted using the survey package in R. P-values were obtained from weighted chi-squared tests for categorical variables and weighted ANOVAs for continuous variables. The reported p-values (α=0.05) for these comparisons highlight variables that are differentially distributed by exposure status and may warrant adjustment as potential confounders in subsequent models. Continuous variables are presented as weighted mean (weighted standard deviation); categorical variables are presented as raw count (weighted percentage).

*Univariate logistic regression*

Univariate logistic regression was performed to preliminarily explore the independent association of each variable with learning difficulty. Variables included: household smoking, gender, age, race/ethnicity, PIR, memory difficulty, attention difficulty, difficulty accepting change, anxiety, and depression (10 variables total). All analyses incorporated NHANES survey weights to correct for bias from the complex sampling design. For clinical interpretability, reference groups for categorical variables were defined based on clinical meaning: ‘no’ for household smoking (lowest exposure), ‘Male’ for gender, and ‘no difficulty’ for cognitive difficulties. Odds ratios for continuous variables (age, PIR) represent the change per one-unit increase.

*Multivariate logistic regression*

Multivariable logistic regression models were used to analyze the association between household smoking environment and learning difficulties. Using complex sampling weights and after handling missing data via multiple imputation, assuming the data were missing at random, four progressively adjusted models were built: Model 1 adjusted for demographics (gender, age, race/ethnicity). Model 2 added socioeconomic status (PIR) to Model 1. Model 3 further added mental health variables (anxiety, depression) to Model 2. Model 4 additionally included cognitive mediators (memory difficulty, attention difficulty, and change acceptance difficulty) to Model 3 to explore potential mediating pathways. All analyses accounted for the complex survey design and applied sampling weights to ensure representativeness of the US population aged 5–17 years. The final estimates for the association between household smoking and learning difficulties, adjusted for all potential confounders (demographics, socioeconomic status, and mental health) while excluding the mediating pathways, were obtained from Model 3.

*Subgroup analysis*

Subgroup analyses were conducted a priori to explore potential heterogeneity in the association between household smoking and learning difficulties across key sociodemographic subpopulations, as these factors may modify vulnerability to environmental exposures. Subgroup analyses were stratified by age (children 5–11 years vs adolescents 12–17 years), gender (male vs female), and race/ethnicity (Hispanic, Non-Hispanic White, Non-Hispanic Black, Other race). Within each subgroup, multivariable logistic regression models were built, adjusting for PIR, anxiety, and depression. All subgroup analyses used survey weights. To test the statistical significance of effect modification between subgroups, likelihood ratio tests compared models containing the interaction term between household smoking and the subgroup variable against models with only main effects.

*Sensitivity analyses*

A complete-case analysis was conducted for sensitivity analysis, including only observations with complete data for all study variables (n=2313; 94.2% of the original sample), in contrast to the primary analysis using multiple imputation. The same multivariable logistic regression models (Models 1–4) as in the primary analysis were applied, using survey weights. To assess potential dose-response relationships and validate the binary classification, we conducted sensitivity analyses using the original three-category variable. This allowed examination of whether an increasing number of household smokers was associated with incrementally higher odds of learning difficulties.

*Mediation analysis*

We employed a mediation analysis method based on bootstrap resampling (n=1000) to explore the mechanism through which household smoking affects learning difficulties within a unified association framework. Using household smoking (smoker present vs no smoker present) as the independent variable and learning difficulties as the dependent variable, the study examined the mediating roles of three cognitive functions: memory difficulties, attention difficulties, and difficulty accepting change. All analyses were adjusted for covariates such as gender, age, poverty ratio, race, anxiety, and depression. The indirect effects were calculated using the difference method, and their statistical significance was assessed via bootstrap confidence intervals. We emphasize that due to the cross-sectional design and as illustrated in Figure 1, mediation estimates represent statistical associations consistent with the hypothesized pathway; temporal precedence (exposure → mediator → outcome) cannot be established, and causal inference is precluded. All statistical analyses were conducted with a significance level of α=0.05.

**Figure 1:**
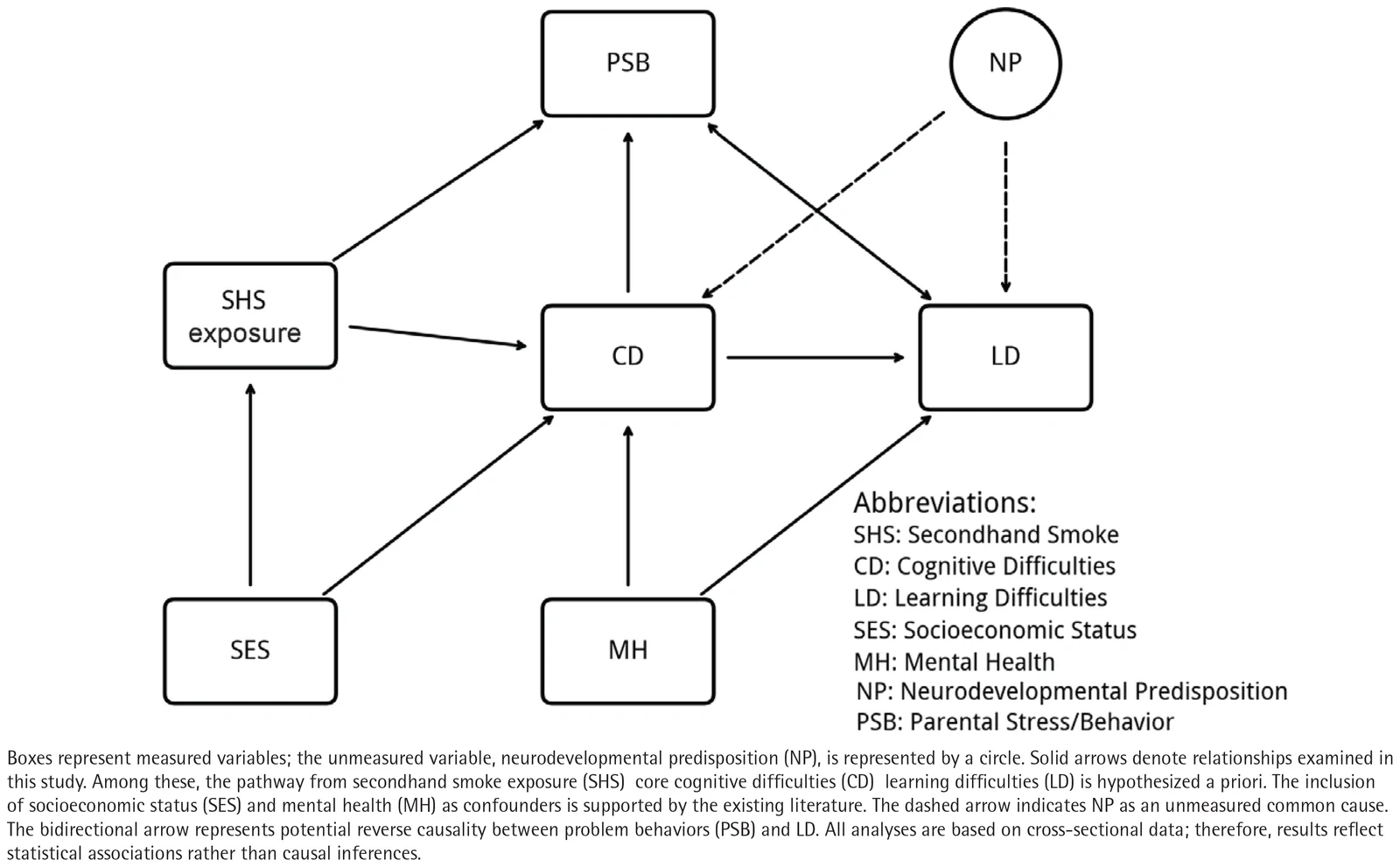
Directed acyclic graph (DAG)

### Ethics

The analysis of NHANES data in this study was conducted under ethical approval granted by the National Center for Health Statistics (NCHS) Research Ethics Review Board. Before participation, written informed consent was acquired from all subjects^13^. Comprehensive information on ethical standards and procedures is accessible via the official NHANES website^13^. Additionally, this research followed the STROBE Guidelines for Observational Studies (Supplementary file Table S2).

## RESULTS

### Descriptive analysis

The weighted prevalence of learning difficulties was 17.4% in the no-smoker household group and 24.6% in the has-smoker(s) household group, a statistically significant difference (p=0.001). Besides learning difficulties, household smoking status was significantly associated with several socioeconomic and cognitive function indicators. PIR showed significant between-group differences (2.8 vs 1.9, p<0.001). Regarding cognitive function, the prevalence of memory difficulty (28.0% vs 19.9%, p=0.001), attention difficulty (22.6% vs 13.4%, p<0.001), and difficulty accepting change (45.2% vs 34.6%, p=0.001) were significantly higher in the has-smoker(s) group. Conversely, anxiety was more frequently reported in the no-smoker group (70.8% vs 62.5%, p=0.031). However, demographic characteristics (age, gender, race/ethnicity) and depressive symptoms showed no significant differences between groups (Table 1).

**Table 1:** Characteristics of study participants by household smoking status, NHANES 2021–2023, United States (N=2455)

*Variable*	*No smokers* *N=1824* *n (%)^a^*	*Has smoker(s)* *N=631* *n (%)^a^*	*p^c^*
**Learning difficulty**			0.001
Yes	330 (17.4)	164 (24.6)	
No	1494 (82.6)	467 (75.4)	
**Gender**			0.3
Female	879 (47.0)	327 (49.4)	
Male	945 (53.0)	304 (50.6)	
**Age** (years), mean (SD)^b^	11.1 (3.7)	11.1 (3.6)	0.7
**Race/ethnicity**			0.7
Mexican American	267 (12.7)	80 (12.4)	
Non-Hispanic Black	253 (11.0)	113 (14.5)	
Non-Hispanic White	732 (46.5)	245 (44.0)	
Other Hispanic	282 (13.8)	82 (11.1)	
Other race	290 (16.1)	111 (18.0)	
**Poverty income ratio**, mean (SD) ^b^	2.8 (1.6)	1.9 (1.4)	<0.001
**Memory difficulty**			0.001
Yes	357 (19.9)	183 (28.0)	
No	1464 (80.1)	448 (72.0)	
**Attention difficulty**			<0.001
Yes	253 (13.4)	149 (22.6)	
No	1570 (86.6)	482 (77.4)	
**Difficulty accepting change**			0.001
Yes	628 (34.6)	292 (45.2)	
No	1195 (65.4)	339 (54.8)	
**Anxiety**			0.031
Yes	1241 (70.8)	394 (62.5)	
No	582 (29.2)	236 (37.5)	
**Depression**			0.9
Yes	914 (50.3)	322 (50.8)	
No	908 (49.7)	309 (49.2)	

a Raw counts, weighted percentages.

b Mean and SD weighted.

c Design-based Kruskal-Wallis test; Pearson’s χ^2^: Rao-Scott adjustment.

Table 2 extends these findings by presenting weighted characteristics stratified by learning difficulty status. In the learning difficulty group, household secondhand smoke exposure was significantly higher (29.9% vs 21.7%, p<0.001), further supporting the association between household smoking environment and learning difficulties. Notably, learning difficulties were significantly associated with lower PIR, as well as higher prevalence of cognitive dysfunction (memory, attention, and difficulty accepting change) and mental health issues (anxiety and depression). Furthermore, in the learning difficulty group, the proportions of cognitive dysfunction were high: memory difficulty at 67.8%, attention difficulty at 43.9%, and difficulty accepting change at 67.0%.

**Table 2:** Characteristics of study participants by learning difficulty status, NHANES 2021–2023, United States (N=2455)

*Variable*	*No difficulty* *N=1961* *n (%)^a^*	*Has difficulty* *N=494* *n (%)^a^*	*p^c^*
**Household smoking status**			<0.001
Has smoker(s)	467 (21.7)	164 (29.9)	
No smokers	1494 (78.3)	330 (70.1)	
**Gender**			0.047
Female	982 (48.5)	224 (43.7)	
Male	979 (51.5)	270 (56.3)	
**Age** (years), mean (SD)^b^	11.0 (3.7)	11.3 (3.7)	0.2
**Race/ethnicity**			0.9
Mexican American	285 (12.8)	62 (11.7)	
Non-Hispanic Black	301 (12.1)	65 (10.4)	
Non-Hispanic White	763 (45.4)	214 (48.1)	
Other Hispanic	293 (13.1)	71 (13.5)	
Other race	319 (16.6)	82 (16.3)	
**Poverty income ratio**, mean (SD)^b^	2.7 (1.6)	2.2 (1.5)	<0.001
**Memory difficulty**			<0.001
Yes	211 (11.0)	329 (67.8)	
No	1749 (89.0)	163 (32.2)	
**Attention difficulty**			<0.001
Yes	175 (8.8)	227 (43.9)	
No	1786 (91.2)	266 (56.1)	
**Difficulty accepting change**			<0.001
Yes	587 (30.0)	333 (67.0)	
No	1374 (70.0)	160 (33.0)	
**Anxiety**			<0.001
Yes	1229 (65.3)	406 (84.3)	
No	731 (34.7)	87 (15.7)	
**Depression**			<0.001
Yes	891 (46.1)	345 (68.7)	
No	1068 (53.9)	149 (31.3)	
**Physical activity** (days/ week)			0.2
0	123 (6.4)	36 (7.4)	
1–2	181 (10.6)	58 (10.1)	
3–4	333 (20.5)	84 (19.4)	
5–7	1078 (62.5)	256 (59.1)	

a Raw counts, weighted percentages.

b Mean and SD weighted.

c Design-based Kruskal-Wallis test; Pearson’s χ^2^: Rao-Scott adjustment.

### Univariate logistic regression analysis

Univariate analysis showed a significant positive association between household smoking and learning difficulties. Children and adolescents living with smoker(s) had 1.54 times the odds of learning difficulties compared to those in smoke-free homes (OR=1.54; 95% CI: 1.24–1.92, p=0.0017). Several psychological and cognitive factors were strongly associated with learning difficulties, with memory difficulty having the strongest association (OR=17.05; 95% CI: 13.33–21.81, p<0.0001), followed by attention difficulty (OR=8.12; 95% CI: 6.09–10.83, p<0.0001) and difficulty accepting change (OR=4.74; 95% CI: 3.72–6.05, p<0.0001). Regarding psychological factors, both anxiety (OR=2.85; 95% CI: 2.08–3.89, p<0.0001) and depression (OR=2.57; 95% CI: 2.07–3.18, p<0.0001) were significantly associated with learning difficulties. Notably, PIR was negatively associated with learning difficulties (OR=0.81; 95% CI: 0.76–0.86, p<0.0001) (Table 3).

**Table 3:** Univariable logistic regression analysis of learning difficulty, NHANES 2021–2023, United States (N=2455)

*Variable*	*Comparison*	*OR (95% CI)*	*p*
**Household smoking**	No smokers (ref.)	1.00	
	Has smoker(s)	1.54 (1.24–1.92)	0.002
**Gender**	Male (ref.)	1.00	
	Female	0.82 (0.68–1.00)	0.067
**Age** (years)	Per year increase	1.02 (0.99–1.06)	0.239
**Race/ethnicity**	Non-Hispanic White (ref.)	1.00	
	Mexican American	0.86 (0.51–1.45)	0.591
	Other Hispanic	0.98 (0.70–1.35)	0.888
	Non-Hispanic Black	0.81 (0.50–1.32)	0.420
	Other race	0.93 (0.62–1.39)	0.731
**Poverty income ratio**	Per unit increase	0.81 (0.76–0.86)	<0.001
**Memory difficulty**	No (ref.)	1.00	
	Yes	17.05 (13.33–21.81)	<0.001
**Attention difficulty**	No (ref.)	1.00	
	Yes	8.12 (6.09–10.83)	<0.001
**Change acceptance difficulty**	No (ref.)	1.00	
	Yes	4.74 (3.72–6.05)	<0.001
**Anxiety**	No (ref.)	1.00	
	Yes	2.85 (2.08–3.89)	<0.001
**Depression**	No (ref.)	1.00	
	Yes	2.57 (2.07–3.18)	<0.001

### Multivariable logistic regression analysis

Multivariable analysis confirmed a significant positive association between household smoking and learning difficulties, robust across adjustment models. After adjusting for demographics (Model 1), the adjusted OR (AOR) was 1.56 (95% CI: 1.26–1.94, p<0.001). Further adjustment for PIR (Model 2) attenuated the association, but it remained significant (AOR=1.36; 95% CI: 1.09–1.70, p=0.006), suggesting socioeconomic factors partially explain the association. Additional adjustment for anxiety and depression (Model 3) slightly increased AOR to 1.43 (95% CI: 1.14–1.79, p=0.002). However, when cognitive mediators (memory difficulty, attention difficulty, and change acceptance difficulty) were added in Model 4, the association became non-significant (AOR=1.05; 95% CI: 0.78–1.40, p=0.764), suggesting these cognitive factors may mediate the relationship between household smoking and learning difficulties (Table 4).

**Table 4:** Multivariable logistic regression analysis of learning difficulty, NHANES 2021–2023, United States (N=2455)

*Comparison*	*Model*	*AOR (95% CI)*	*p*
**Has smoker(s)**	Model 1	1.56 (1.26–1.94)	0.002
	Model 2	1.36 (1.09–1.70)	0.031
	Model 3	1.43 (1.14–1.79)	0.002
	Model 4	1.05 (0.78–1.40)	0.764

AOR: adjusted odds ratio. Model 1: adjusted for household smoking, gender, age and race/ethnicity. Model 2: as for Model 1 plus poverty income ratio. Model 3: as for Model 2 plus anxiety and depression. Model 4: as for Model 3 plus memory difficulty, attention difficulty and change acceptance difficulty.

### Subgroup analysis

Subgroup analysis by age showed a stronger association in adolescents (12–17 years: AOR=1.48; 95% CI: 1.04–2.10, p=0.028) than in children (5–11 years: AOR=1.33; 95% CI: 1.00–1.77, p=0.047), but the interaction was not significant (p=0.652). By gender, the association was significant in females (AOR=1.62; 95% CI: 1.10–2.38, p=0.015) but not in males (AOR=1.27; 95% CI: 0.88–1.83, p=0.200), though the interaction was non-significant (p=0.531). By race/ethnicity, associations were not statistically significant for Hispanic (AOR=1.59; 95% CI: 0.90–2.82, p=0.113), Non-Hispanic White (AOR=1.19; 95% CI: 0.77–1.85, p=0.439), Non-Hispanic Black (AOR=1.10; 95% CI: 0.57–2.09, p=0.782), or Other races (AOR=1.71; 95% CI: 0.97–3.03, p=0.065); the interaction was non-significant (p=0.814) (Table 5).

**Table 5:** Subgroup analysis of the association between household smoking and learning difficulties, stratified by age, gender, and race/ethnicity, NHANES 2021–2023, United States (N=2455)

*Subgroup*	*AOR (95% CI)*	*p*	*p for interaction*
Adolescents (12–17 years)	1.48 (1.04–2.10)	0.028	0.652
Children (5–11 years)	1.33 (1.00–1.77)	0.047	
Female	1.62 (1.10–2.38)	0.015	0.531
Male	1.27 (0.88–1.83)	0.200	
Hispanic	1.59 (0.90–2.82)	0.113	0.814
Non-Hispanic Black	1.10 (0.57–2.09)	0.782	
Non-Hispanic White	1.19 (0.77–1.85)	0.439	
Other race	1.71 (0.97–3.03)	0.065	

AOR: adjusted odds ratio. Adjusted for poverty income ratio, anxiety, and depression.

### Sensitivity analysis

The complete case sensitivity analysis yielded results consistent with the primary analysis. The association remained significant in Models 1–3 (Model 1: AOR=1.65; 95% CI: 1.32–2.08, p=0.002; Model 2: AOR=1.35; 95% CI: 1.08–1.69, p=0.031; and Model 3: AOR=1.43; 95% CI: 1.14–1.79, p=0.026). Importantly, Model 4 showed attenuation to non-significance (AOR=1.05; 95% CI: 0.78–1.40, p=0.792), mirroring the pattern observed in the primary analysis. Effect directions (OR>1) and significance (p<0.05) were consistent, confirming the robustness of the findings against missing data handling methods and supporting the potential mediating role of cognitive factors (Supplementary file Table S3). Additionally, sensitivity analyses using the three-category household smoking variable revealed an initial dose-response pattern: compared to no household smokers, children with 1 smoker had 47% higher odds of learning difficulties (AOR=1.47; 95% CI: 1.21–1.79), and those with ≥2 smokers had 79% higher odds (AOR=1.79; 95% CI: 1.17–2.74; p for trend=0.007). This gradient attenuated with progressive adjustment, and in the fully adjusted model, neither 1 smoker (AOR=1.15; 95% CI: 0.76–1.75, p=0.508) nor ≥2 smokers (AOR=0.85; 95% CI: 0.55–1.31, p=0.455) showed significant associations (Supplementary file Table S4).

### Mediation analysis

The mediation analysis results revealed a significant total effect between household smoking and learning difficulties (β=0.370, 95% CI: 0.135–0.630). All three cognitive function mediators demonstrated significant mediating effects. The mediating effect of memory difficulties was 0.192 (95% CI: 0.055–0.335, p=0.006), accounting for 52.1% of the total effect. The mediating effect of attention difficulties was 0.164 (95% CI: 0.070–0.265, p<0.001), accounting for 44.3% of the total effect. The mediating effect of difficulty accepting change was 0.135 (95% CI: 0.074–0.199, p<0.001), accounting for 36.4% of the total effect. The total indirect effect of the three mediators was 0.491 (Figure 2).

**Figure 2:**
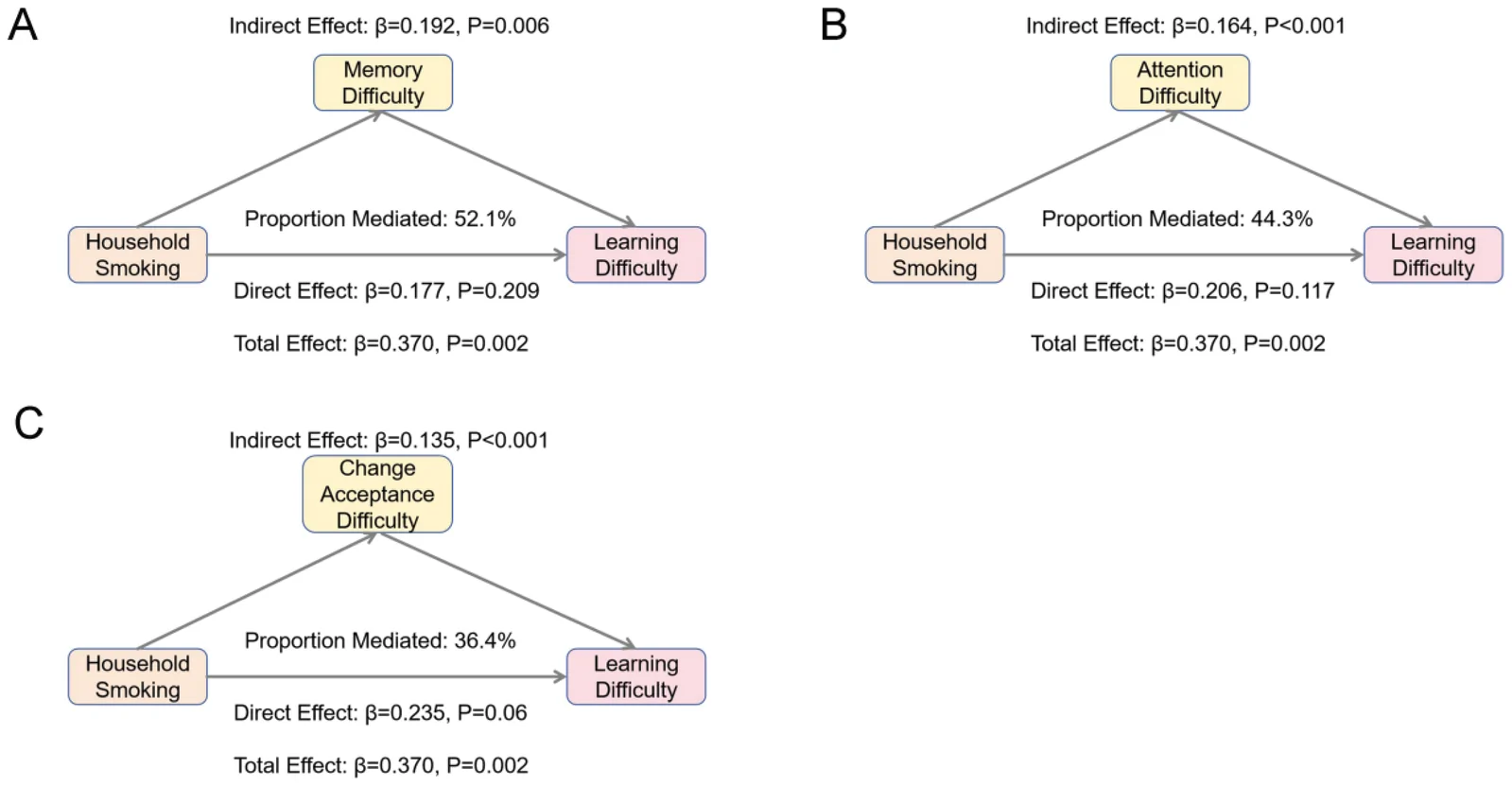
Mediation analysis: A) Memory difficulty; B) Attention difficulty; C) Change acceptance difficulty. Adjusted covariates: gender, age, race/ethnicity, poverty income ratio, anxiety, and depression

## DISCUSSION

The presence of smoker(s) in the household was independently associated with a significantly higher likelihood of learning difficulties. This association was primarily mediated through impairments in core cognitive functions, particularly attention, memory, and adaptability to change. These findings not only provide robust, recent population-based evidence for the negative impact of household secondhand smoke exposure on child neurodevelopment and learning capacity but also deepen our understanding of the ‘environmental toxin–cognitive function–academic performance’ pathway.

### Household smoking environment as an independent correlate of learning difficulties

The core finding is the robust, significant positive association between household smoking and learning difficulties, which persisted after adjusting for a range of important potential confounders including demographics, socioeconomic status (PIR), and mental health symptoms. In our conceptual model, we treated PIR as a confounder, consistent with the perspective that socioeconomic disadvantage is a foundational determinant that precedes and influences both the likelihood of household smoking and child development outcomes^15,16^. This association demonstrated remarkable stability across multiple analytical approaches, as evidenced by the consistent results in the primary multiple imputation analysis, the complete-case sensitivity analysis, and the alternative categorization sensitivity analysis. The stability of this association across different methodological approaches strengthens the credibility of our findings and suggests a genuine biological relationship rather than a methodological artifact.

Notably, household socioeconomic status played a partial mediating role. The association weakened when PIR was added, indicating that household smoking often coexists with lower socioeconomic status, itself a known risk factor for learning difficulties^17,18^. This pattern aligns with the social determinants of health framework, which posits that health risks often cluster in disadvantaged populations. Families with lower socioeconomic status may face multiple concurrent challenges, including higher smoking prevalence, limited access to educational resources, and increased environmental stressors^19-21^, all of which can cumulatively impact children’s cognitive development and academic performance. We acknowledge the potential for bidirectional relationships, wherein smoking may impose financial strain^22^. However, our analytical focus was on estimating the association between household smoking and learning difficulties after accounting for the overarching context of socioeconomic position; hence, our modeling choice to treat PIR as a confounder.

However, the persistent significance after adjustment strongly suggests that the harm of secondhand smoke exposure extends beyond the additive effects of socioeconomic disadvantage, possessing its own unique neurotoxic impact. The biological plausibility of this direct effect is supported by growing evidence from toxicological and neuroimaging studies. Secondhand smoke contains over 7000 chemicals, including neurotoxicants such as nicotine, lead, and carbon monoxide, which can directly affect the developing brain^23^. Nicotine, in particular, is known to disrupt cholinergic signaling pathways crucial for attention, learning, and memory formation^24^. Furthermore, emerging research suggests that secondhand smoke exposure may induce neuroinflammation and oxidative stress, potentially leading to structural and functional changes in brain regions involved in cognitive processing, such as the prefrontal cortex and hippocampus^25,26^.

### Cognitive impairment as a key mediating mechanism

The most innovative finding is the quantification, through mediation analysis, of the central mediating roles of memory difficulty, attention difficulty, and difficulty accepting change. The mediation analysis demonstrated that indirect effects consistently exceeded direct effects, indicating that the impact of household smoking on learning ability operates primarily through impairment of these basic cognitive processes, while also allowing for additional pathways.

This finding provides population-scale empirical support for the hypothesized nicotine neurotoxicity mechanisms discussed in the introduction. Prior literature indicates that nicotine, as a neuroactive substance, can disrupt the normal function of nAChRs, affecting brain regions critical for higher cognition like the prefrontal cortex and hippocampus^27^. Our results resonate with targeted studies: Korean researchers found that attention deficits observed in TSE-exposed children on the CPT mediated their learning problems^7^; studies on verbal memory also suggest tobacco use is linked to slowed learning and retrieval^9^. Our study integrates multiple cognitive domains within a single analytical framework, demonstrating their collective role as a key statistical pathway.

Memory difficulty demonstrated the highest mediation proportion, highlighting its pivotal role in the pathway from household smoking to learning difficulties. This finding underscores the fundamental importance of encoding, storage, and retrieval processes in learning, as impairments in these memory functions may directly compromise a child’s ability to acquire and consolidate new knowledge. Attention difficulty also showed a substantial proportion of mediation, likely because attention serves as the critical gateway to learning, and its impairment directly reduces information-processing efficiency^28^. The mediating role of difficulty accepting change suggests secondhand smoke exposure might also affect cognitive flexibility and emotional/behavioral regulation, making it harder for children to adapt to changing tasks and demands in learning, thereby exacerbating difficulties^29,30^.

### Subgroup analysis

Subgroup analysis suggested potentially stronger associations in adolescents (12–17 years) and female children, although interactions were not statistically significant. This might indicate increased sensitivity to the neurotoxic effects of tobacco smoke during adolescence, a period of heightened academic demands and brain remodeling^31^. The higher risk in females could relate to gender-specific neurodevelopmental trajectories or differential susceptibility to socio-environmental stressors, warranting further investigation^32^.

An unexpected finding was the higher reported rate of anxiety in non-smoking households in descriptive analysis. This contradicts common knowledge and most literature^10,11^, potentially due to several reasons. Firstly, the single-item anxiety measure used in this study likely lacks the sensitivity and specificity of validated clinical instruments, limiting nuanced assessment. Secondly, this finding could reflect residual confounding or reporting bias, e.g. parents in non-smoking households might be more aware of or less stigmatized in reporting their children’s internalizing symptoms. Thirdly, there is a possibility that household smoking itself could mask or alter the phenotypic expression of anxiety^33^. This paradox highlights the complexity of mental health assessment in observational studies and requires verification in future research using more refined tools.

### Strengths and limitations

This study leverages the nationally representative, high-quality NHANES 2021–2023 data to investigate this public health issue. The use of survey weights in our analyses enhances the generalizability of the findings to US children and adolescents aged 5–17 years. The methodological approach, including rigorous multivariable adjustments, sensitivity analyses, and formal mediation analysis, strengthens the study’s robustness and supports its mechanistic interpretation.

Limitations must be acknowledged. Firstly, the sample size was determined by data availability in the NHANES 2021–2023 cycles rather than by a prospective power calculation, a limitation that should be considered when interpreting the results. Secondly, the cross-sectional design precludes establishing definitive causality. Although we controlled for a range of confounders guided by theory and DAGs, residual confounding by unmeasured or unavailable factors cannot be entirely ruled out. Specifically, important potential confounders such as parental education and mental health, household crowding, environmental pollutants, child’s screen time, sleep quality, and particularly prenatal secondhand smoke exposure were not available in the NHANES dataset for our analytical sample. Their omission may have influenced the observed associations and should be considered when interpreting the results. Thirdly, reliance on single-item, self- or proxy-reported measures is a key limitation, as these items are not clinically validated and are susceptible to recall and social desirability biases, potentially leading to misclassification. The NHANES cycles we used did not include more comprehensive, validated scales for the core constructs in our study, constraining our analysis to these proxy measures. Fourthly, while mediation analysis suggests a key role for cognitive function, the fundamental assumption of temporal sequence (exposure → mediator → outcome) cannot be verified in cross-sectional data, and our estimates require cautious interpretation as statistical associations. Ideally, longitudinal validation is needed. Furthermore, this study focused on three core cognitive mediators; future research should investigate other potential pathways, such as mental health symptoms or biological markers, in the association between secondhand smoke exposure and learning difficulties. Finally, dichotomizing learning and cognitive difficulty variables ensured statistical power but may have lost information on severity gradients.

## CONCLUSIONS

This study, based on a large nationally representative sample, confirms that household smoking environment is independently associated with learning difficulties in children and adolescents, and this association is primarily mediated by tobacco smoke-induced impairments in core cognitive functions like attention, memory, and adaptability to change. From a public health policy perspective, these findings highlight the need for further longitudinal and interventional studies to confirm the observed associations and to establish whether creating smoke-free home environments and implementing tobacco control interventions could effectively reduce learning difficulties in children and adolescents.

## Supplementary Material



## Data Availability

The data supporting this research are available from the authors on reasonable request.
