# Supplementary material for: Household secondhand smoke exposure and learning difficulties in children and adolescents: A cross-sectional study on the mediating roles of attention, memory, and adaptability
Source: Tob Induc Dis. 2026 Oct 31;24:10.18332/tid/226713. doi: 10.18332/tid/226713 (PMC13733296; doi:10.18332/tid/226713)
Supplement: Supplementary file 1 [file TID-24-204-s1.pdf]

Tobacco Induced Diseases

Supplementary file

© 2026 Lu Z. and Ye B.

DOI:
10.18332/tid/226713

The content has been provided by the author(s) and has not been reviewed, verified, or endorsed by European Publishing. It may not have undergone peer review. The views, opinions, and recommendations expressed are solely those of the author(s) and do not necessarily reflect the position of European Publishing. European Publishing accepts no responsibility or liability for any consequences arising from the use of, or reliance on, this content.

Supplementary Table S1. Description of NHANES (2021-2023) variables used in the analysis.

Category	NHANES Variable	Description	Original Codes / Values	Analytical Coding / Handling
Dependent Variable	FNQ120	Learning difficulties	1: No difficulty<br>2: Some difficulty<br>3: A lot of difficulty<br>4: Cannot do at all	Binary: 1 (No difficulty) vs. 2/3/4 (Has difficulty).
Primary Exposure	SMD460	Numbers of people in the household who smoke	0: No one in household is a smoker<br>1: 1 household member is a smoker<br>2: 2 or more household members are smokers	Binary: 0 (No smoker) vs. 1/2 (Has smoker(s)).
Putative Mediators	FNQ170	Memory difficulties	1: No difficulty<br>2: Some difficulty<br>3: A lot of difficulty<br>4: Cannot do at all	Binary: 1 (No difficulty) vs. 2/3/4 (Has difficulty).
	FNQ180	Difficulty concentrating/ remembering	1: No difficulty<br>2: Some difficulty<br>3: A lot of difficulty<br>4: Cannot do at all	Binary: 1 (No difficulty) vs. 2/3/4 (Has difficulty).
	FNQ190	Difficulty accepting changes in routine	1: No difficulty<br>2: Some difficulty<br>3: A lot of difficulty<br>4: Cannot do at all	Binary: 1 (No difficulty) vs. 2/3/4 (Has difficulty).
Covariates	RIAGENDR	Gender	1: Male<br>2: Female	Categorical.
	RIDAGEYR	Age in years at screening	5-17	Continuous.
	RIDRETH1	Race/Hispanic origin	1: Mexican American<br>2: Other Hispanic<br>3:Non-Hispanic White<br>4:Non-Hispanic Black<br>5: Other Race	Categorical.
	INDFMPIR	Family PIR (ratio of family income to poverty)	0.00 - 5.00	Continuous.
	FNQ140	Feel worried, nervous, or	1: Daily<br>2: Weekly<br>3: Monthly	Binary: 1-4 (Yes, has anxiety) vs. 5 (No anxiety).

	anxious	4: A few times a year<br>5: Never	
FNQ150	Feel depressed	1: Daily<br>2: Weekly<br>3: Monthly<br>4: A few times a year<br>5: Never	Binary:1-4 (Yes, has anxiety) vs. 5 (No anxiety).
PAQ706	Numbers of days physically active at least 60 min (past 7 days)	0-7: Number of days	Categorical:<br>0: 0 days<br>1-2: 1-2 days<br>3-4: 3-4 days<br>5-7: 5-7 days.

Supplementary Table S2. STROBE Statement—checklist of items that should be included in reports of observational studies

	Item No	Recommendation	Page No
**Title and abstract**	1	(*a*) Indicate the study's design with a commonly used term in the title or the abstract	1
		(*b*) Provide in the abstract an informative and balanced summary of what was done and what was found	1
**Introduction**			
Background/rationale	2	Explain the scientific background and rationale for the investigation being reported	2
Objectives	3	State specific objectives, including any prespecified hypotheses	3
**Methods**			
Study design	4	Present key elements of study design early in the paper	3
Setting	5	Describe the setting, locations, and relevant dates, including periods of recruitment, exposure, follow-up, and data collection	3
Participants	6	*Cross-sectional study*—Give the eligibility criteria, and the sources and methods of selection of participants	3
Variables	7	Clearly define all outcomes, exposures, predictors, potential confounders, and effect modifiers. Give diagnostic criteria, if applicable	3-4
Data sources/ measurement	8*	For each variable of interest, give sources of data and details of methods of assessment (measurement). Describe comparability of assessment methods if there is more than one	3-4

		group	
Bias	9	Describe any efforts to address potential sources of bias	3-6
Study size	10	Explain how the study size was arrived at	3
Quantitative variables	11	Explain how quantitative variables were handled in the analyses. If applicable, describe which groupings were chosen and why	3-4
Statistical methods	12	(*a*) Describe all statistical methods, including those used to control for confounding	4-6
		(*b*) Describe any methods used to examine subgroups and interactions	5
		(*c*) Explain how missing data were addressed	3
		(*e*) Describe any sensitivity analyses	5

Results

Participants	13*	(a) Report numbers of individuals at each stage of study—eg numbers potentially eligible, examined for eligibility, confirmed eligible, included in the study, completing follow-up, and analysed	3
		(b) Give reasons for non-participation at each stage	3
		(c) Consider use of a flow diagram	n/a
Descriptive data	14*	(a) Give characteristics of study participants (eg demographic, clinical, social) and information on exposures and potential confounders	Table 1, Table 2
		(b) Indicate number of participants with missing data for each variable of interest	3
		(c) *Cohort study*—Summarise follow-up time (eg, average and total amount)	n/a
Outcome data	15*	*Cohort study*—Report numbers of outcome events or summary measures over time	n/a
		Case-control study—Report numbers in each exposure category, or summary measures of exposure	n/a
		Cross-sectional study—Report numbers of outcome events or summary measures	Table 2
Main results	16	(*a*) Give unadjusted estimates and, if applicable, confounder-adjusted estimates and their precision (eg, 95% confidence interval). Make clear which confounders were adjusted for and why they were included	Table 4
		(*b*) Report category boundaries when continuous variables were categorized	n/a
		(*c*) If relevant, consider translating estimates of relative risk into absolute risk for a meaningful time period	n/a
Other analyses	17	Report other analyses done—eg analyses of subgroups and interactions, and sensitivity analyses	Table 5, Table 6, Table 7, Figure 2

Discussion

Key results	18	Summarise key results with reference to study objectives	15
Limitations	19	Discuss limitations of the study, taking into account sources of potential bias or imprecision. Discuss both direction and magnitude of any potential bias	17
Interpretation	20	Give a cautious overall interpretation of results considering objectives, limitations, multiplicity of analyses, results from similar studies, and other relevant evidence	15-17
Generalisability	21	Discuss the generalisability (external validity) of the study results	n/a

Other information

Funding	22	Give the source of funding and the role of the funders for the present study and, if applicable, for the original study on which the present article is based	No funding was received

*Give information separately for cases and controls in case-control studies and, if applicable, for exposed and unexposed groups in cohort and cross-sectional studies.

Note: An Explanation and Elaboration article discusses each checklist item and gives methodological background and published examples of transparent reporting. The STROBE checklist is best used in conjunction with this article (freely available on the Web sites of PLoS Medicine at http://www.plosmedicine.org/, Annals of Internal Medicine at http://www.annals.org/, and Epidemiology at http://www.epidem.com/). Information on the STROBE Initiative is available at www.strobe-statement.org.

Supplementary Table S3. Sensitivity Analysis 1: Complete Case Analysis, NHANES 2021–2023 (N = 2,313), United States

Comparison	Models	Odds Ratio (95% CI)	P Value
Has smoker(s) vs No smokers	**Model 1**	1.66 (1.32-2.09)	0.002
	**Model 2**	1.36 (1.09-1.70)	0.031
	**Model 3**	1.44 (1.14-1.80)	0.026
	**Model 4**	1.05 (0.78-1.40)	0.792

Note:

CI = Confidence Interval;

Model 1: Household smoking + Gender + Age + Race/Ethnicity

Model 2: Model 1 + Poverty income ratio

Model 3: Model 2 + Anxiety + Depression

Model 4: Model 3 + Memory difficulty + Attention difficulty + Change acceptance difficulty

Complete cases sample size: 2310 (94.1 % of total sample).

Supplementary Table S4. Sensitivity Analysis 2: Household Smoking as a Three-Category Variable, NHANES 2021–2023 (N = 2,455), United States

Models	Comparison	Odds Ratio (95% CI)	P Value
**Model 1**	**1 smoker vs No smokers**	1.47 (1.21-1.79)	<0.001
	**≥2 smokers vs No smokers**	1.79 (1.17-2.74)	0.007
**Model 2**	**1 smoker vs No smokers**	1.39 (1.14-1.69)	0.001
	**≥2 smokers vs No smokers**	1.30 (0.80-2.12)	0.281
**Model 3**	**1 smoker vs No smokers**	1.47 (1.17-1.85)	<0.001
	**≥2 smokers vs No smokers**	1.34 (0.87-2.08)	0.187
**Model 4**	**1 smoker vs No smokers**	1.15 (0.76-1.75)	0.507
	**≥2 smokers vs No smokers**	0.85 (0.55-1.31)	0.455

Note:

CI = Confidence Interval;

Model 1: Household smoking + Gender + Age + Race/Ethnicity;

Model 2: Model 1 + Poverty income ratio;

Model 3: Model 2 + Anxiety + Depression;

Model 4: Model 3 + Memory difficulty + Attention difficulty + Change acceptance difficulty;

Sample size：No smokers: 1824; 1 smoker: 435; ≥2 smoker: 196.
